# Impact of COVID-19 related home confinement measures on the lifestyle, body weight, and perceived glycemic control of diabetics

**DOI:** 10.1016/j.metop.2021.100144

**Published:** 2021-10-29

**Authors:** Muna Abed Alah, Sami Abdeen, Vahe Kehyayan, Iheb Bougmiza

**Affiliations:** a Community Medicine Department, Hamad Medical Corporation (HMC), Doha, Qatar; b University of Calgary in Qatar, Doha, Qatar; c Community Medicine Department, Primary Health Care Corporation (PHCC), Doha, Qatar; d Community Medicine Department, College of Medicine, Sousse University, Tunisia

**Keywords:** COVID-19, Diabetes, Lifestyle, Glycemic control, Diet, Physical activity

## Abstract

**Background:**

People with Diabetes Mellitus are at high risk of encountering COVID-19 infection and are more vulnerable to the negative repercussions of this infection. In this study we aimed to explore the impact of COVID-19 related home confinement measures on physical activity, dietary habits, body weight and perceived glycemic control of adults with type 2 Diabetes Mellitus (T2DM) in Qatar.

**Methods:**

A cross sectional web-based survey was conducted between January and February 2021 targeting adults ≥18 years with T2DM.

**Results:**

Over 40% of the participants reported unhealthy dietary changes. We found a significant increase in the sitting/reclining, and screen times. One third of the participants reported weight gain, while one fifth reported poorer glycemic control since the start of home confinement measures. We found that reporting at least one unhealthy dietary change (p < 0.001) and being a female (p = 0.002) were significantly associated with reporting greater weight gain. Participants who reported five unhealthy dietary behaviours were more than seven times more likely to perceive poorer glycemic control during home confinement measures compared to those who did not report any unhealthy changes (OR: 7.27, 95%CI 1.60–33.5, p = 0.011).

**Conclusion:**

Adults with T2DM experienced adverse lifestyle changes during COVID-19 related home confinement measures. Further research is needed to investigate the persistence of such changes in the post pandemic era.

## Highlights

• One third of the participants reported weight gain.
• Participants reported a significant increase in each of the sitting/reclining and screen times.
• The perceived glycemic control varied among the participants.

## 1 Introduction

A growing body of evidence has shown that people with chronic diseases such as Diabetes Mellitus (DM) are at high risk of getting infected with SARS-CoV2 [[1], [2], [3]]. DM is associated with increased mortality and severity of disease in COVID-19 pneumonia [4]. On the other hand, movement restrictions and lockdown measures imposed during the COVID-19 pandemic resulted in deteriorated glycemic control among diabetic patients [5,6]. Reasons behind such deterioration include interruption of medical services with limited access to healthcare, treatment delays, low patients’ compliance with medications and adopting unhealthy lifestyle habits during the lockdown measures including reduced physical activity especially with closures of gyms and shopping malls, shifting to work from home, and unhealthy dietary habits resulting in weight gain [[6], [7], [8]]. Nutritional and behavioral interventions are of paramount importance and maybe more important than medical interventions to achieve better glycemic control during the unprecedented crises of the COVID-19 pandemic. Improving the metabolic health is crucial to win the fight against COVID-19 and any other potential future outbreaks [9]. In this study we aimed to explore the impact of COVID-19 related home confinement measures on physical activity, dietary habits, body weight and perceived glycemic control of adults with type 2 Diabetes Mellitus (T2DM) in Qatar.

## 2 Methods

We conveniently invited adults ≥18 years with T2DM to take part in an online web-based survey that was adapted from other validated and reliable questionnaires, which we then translated into three other languages (Arabic, Urdu, and Malayalam) [10,11], between January and February 2021. The link to the online version of the questionnaire was posted on social media platforms and was circulated through WhatsApp groups (Supplementary file 1).

We collected sociodemographic characteristics of participants such as age, gender, nationality, marital status, employment status, educational level, and comorbidities. The questionnaire also assessed changes in dietary habits (a list of healthy and unhealthy dietary behaviours), physical activity including changes in time spent in exercise (regardless of the type or intensity of exercise), sitting/reclining, and screen times (expressed as hours/day before and during home confinement), weight gain (amount of weight gain during home confinement expressed as weight gain categories (no change, >3 kg, 3–6 kg, 7–10 kg, > 10 kg), the perceived glycemic control (expressed as less controlled, more controlled, or the same) and the reasons behind poorer glycemic control. An ethical approval was obtained from the institutional review board (IRB) of Hamad Medical Corporation (MRC 01-20-838). Taking the survey implied informed consent.

### 2.1 Data analysis

We used the Statistical Package for the Social Sciences (SPSS) version 26 to analyse the data. Descriptive statistics were presented as frequencies and percentages for categorical variables. The normality of continuous variables was tested using Shapiro Wilk test, and the univariable analysis using nonparametric Mann-Whitney U, and Kruskal Wallis tests was used to compare ordinal and not normally distributed continuous variables between different subgroups. We used the Wilcoxon Signed Rank test to test the differences in screen, sitting/reclining, and exercise times as expressed before and during the home confinement measures. We carried out an ordinal logistic regression to determine the predictors of weight gain considering weight gain categories as a dependent ordinal variable, and a multiple logistic regression analysis to determine the predictors of glycemic control among participants taking into consideration the glycemic control status as binary dependent variable (less controlled vs more controlled or the same combined). Goodness of Fit was assessed using Pearson and Deviance tests for ordinal regression and Hosmer-Lemeshow test for logistic regression. The variables included in the regression models were selected based on clinical relevance (based on risk factors established in the literature), and statistical significance (variables with P values of <0.25 on the univariable analysis were included in the model). The associations between risk factors and outcomes are presented as adjusted odds ratios (ORs) and 95% confidence intervals (95%CIs). P-values of less than 0.05 were considered significant.

## 3 Results

A total of 171 diabetics completed the survey. The majority (40.4%) were between 35 and 44 years of age, males (68.4%), non- Arabs (90.1%). A total of 148 (86.5%) participants were employed with 78 (52.7%) of them worked from home during home confinement measures. About one quarter (25.7%) of the participants had other comorbidities beside T2DM, most commonly hypertension (Table 1).

**Table 1:** Sociodemographic profiles and primary outcomes of the participants.

Variable			No (%)
**Age**	18–34		39 (22.8)
	35–44		69 (40.4)
	45–54		47 (27.5)
	≥55		16 (9.4)
**Gender**	Male		117 (68.4)
	Female		54 (31.6)
**Nationality**^a^	Arab		154 (90.1)
	Non-Arab		17 (9.9)
**Highest degree of education**	No formal education		1 (0.6)
	High school diploma		30 (17.5)
	College or Higher		134 (78.4)
	Vocational training		6 (3.5)
**Employment status**	Employed		148 (86.5)
	Not employed		23 (13.5)
**Marital status**	Married		156 (91.2)
	Not married		15 (8.8)
**Comorbid disease/s**^b^	No		127 (74.3)
	Yes		44 (25.7)
**Number of unhealthy dietary changes**	None		59 (34.5)
	Yes	1	29 (17.0)
		2	28 (16.4)
		3	19 (11.1)
		4	23 (13.5)
		5	13 (7.6)
**Weight gain categories**	No weight gain		105 (61.4)
	Less than 3 Kg		20 (11.7)
	3-6 Kg		38 (22.2)
	7 Kg or more		8 (4.7)
**Perceived glycemic control**	Less controlled		36 (21.1)
	The same		97 (56.7)
	More controlled		38 (22.2)
**Exercise time difference**^c^**(Hours/day)**			0.00 (1.25)
**Sitting/reclining time difference**^c^**(Hours/day)**			1.66 (3.97)
**Screen time Difference**^c^**(Hours/day)**			1.83 (3.43)

a More than 20 different nationalities were reported.

b The most commonly reported comorbidity was Hypertension.

c Reported as Mean (SD).

The participants reported several unhealthy dietary changes most commonly eating larger quantities of food (46.2%), eating more fat rich food (43.3%), and more sugar and/or sweetened food or beverages (40.4%). On the other hand, 90.6%, and 82.5% of the participants reported healthier changes such as depending more on home cooking and eating more fruits and vegetables, respectively. Upon assessing the changes in physical activity, we found a significant increase in each of the sitting/reclining (1.66 h/day mean increase, 95%CI:1.06–2.26), and screen times (1.83 h/day mean increase, 95%CI:1.31–2.35), with p < 0.001. On the other hand, we did not find any significant change in the exercise time. About one third (38.6%) of the participants reported some weight gain since the start of home confinement measures with more than half of them (57.6%) reported an increase of 3–6 kg in their body weight. Of all participants, 36 (21.1%), 38 (22.2%), and 97(56.7%) admitted that their blood sugar readings became less controlled, more controlled, and the same respectively. The majority attributed their less controlled readings to less healthy dietary choices, more sedentary behaviours, and more stress during COVID-19 related home confinement measures. As shown in Table 2, participants who reported at least one unhealthy dietary change were 13 times more likely to report greater weight gain than those who did not report such changes (OR 13:10, 95%CI 4.56–37.6, p < 0.001). Females were more than 4 times more likely to report greater weight gain compared to males (OR 4.35, 95%CI:1.71–11.05, p = 0.002). Similarly, those who were previously active (used to go to the gym before home confinement measures) were more likely to fall in higher weight gain categories (OR: 2.59, 95%CI 1.06–6.36, p = 0.037). Upon assessing the predictors of glycemic control, participants who reported five unhealthy dietary behaviours were more than seven times more likely to perceive poorer glycemic control during home confinement measures compared to those who did not report any unhealthy changes (OR: 7.27, 95%CI 1.60–33.5, p = 0.011), Table 3.

**Table 2:** Determinants and predictors of weight gain during COVID-19 related home confinement among adults with type 2 diabetes mellitus.

Variable		No (%) of participants in each weight gain category			Univariable analysis *p*-value	Multivariable analysis^a^	
		No increase in weight	Less than 3 kg	3 kg or more		AOR (95%CI)	*p*-value
**Age**	18–34	20 (51.3)	4 (10.3)	15 (38.5)	**0.046**	4.30 (061–30.16)	0.143
	35–44	40 (58.0)	8 (11.6)	21 (30.4)		4.51 (0.65–31.34)	0.127
	45–54	31 (66.0)	7 (14.9)	9 (19.1)		5.00 (0.77–32.5)	0.093
	≥55	14 (87.5)	1 (6.3)	1 (6.3)		1 [Reference]	
**Gender**	Male	84 (71.8)	12 (10.3)	21 (17.9)	**<0.001**	1 [Reference]	
	Female	21 (38.9)	8 (14.8)	25 (46.3)		4.35 (1.71–11.05)	**0.002**
**Nationality**	Arab	6 (35.3)	2 (11.8)	9 (52.9)	**0.011**	1.81 (0.50–6.54)	0.365
	Non-Arab	99 (64.3)	18 (11.7)	37 (24.0)		1 [Reference]	
**Highest degree of education**	No formal education	1 (100.0)	0 (0.0)	0 (0.0)	0.259		>0.999
	High school diploma	19 (63.3)	6 (20.0)	5 (16.7)		0.14 (0.15–1.36)	0.091
	College or Higher	83 (61.9)	14 (10.4)	37 (27.6)		0.12 (0.14–1.04)	0.055
	Vocational training	2 (33.3)	0 (0.0)	4 (66.7)		1 [Reference]	
**Employment status**	Employed	99 (66.9)	14 (9.5)	35 (23.6)	**<0.001**	1.04 (0.33–3.24)	0.950
	Not employed	6 (26.1)	6 (26.1)	11 (47.8)		1 [Reference]	
**Marital status**	Married	100 (64.1)	17 (10.9)	39 (25.0)	**0.023**	0.46 (0.12–1.73)	0.250
	Not married	5 (33.3)	3 (20.0)	7 (46.7)		1 [Reference]	
**Comorbid disease/s**	No	76 (59.8)	17 (13.4)	34 (26.8)	0.628	0.60 (0.22–1.64)	0.315
	Yes	29 (65.9)	3 (6.8)	12 (27.3)		1 [Reference]	
**Being previously active (attending gym regularly**	Yes	13 (40.6)	7 (21.9)	12 (37.5)	**0.016**	2.59 (1.05–6.36)	**0.037**
	No	92 (66.2)	13 (9.4)	34 (24.5)		1 [Reference]	
**Unhealthy dietary change**	No	53 (89.8)	4 (6.8)	2 (3.4)	**<0.001**	1 [Reference]	
	Yes (at least one unhealthy change)	52 (46.4)	16 (14.3)	44 (39.3)		13.10 (4.56–37.61)	**<0.001**
**Mean time difference for each weight gain category**							
**Exercise time difference**^†^**(Hours/day)**		0.10	0.30	−0.34	0.074	1.06 (0.78–1.44)	0.719
**Sitting/reclining time difference**^†^**(Hours/day)**		1.18	1.55	2.80	**0.021**	1.13 (1.01–1.26)	**0.038**
**Screen time Difference**^†^**(Hours/day)**		1.55	1.15	2.76	0.063	0.88 (0.77–1.02)	0.087

Abbreviations: AOR adjusted odds ratio; CI, confidence interval.

a Ordinal logistic regression model was used.

**Table 3:** Determinants and predictors of the perceived glycemic control during COVID-19 related home confinement among adults with type 2 diabetes mellitus.

Variable			Less Controlled N (%)	Univariable analysis *p*-value*	Multivariable analysis	
					AOR (95%CI)	*p*-value
**Age**	18–34		8 (20.5)	0.229	1.32 (0.11–16.00)	0.826
	35–44		19 (27.5)		3.07 (0.29–32.81)	0.353
	45–54		8 (17.0)		3.22 (0.29–36.36)	0.344
	≥55		1 (6.3)		1 [Reference]	
**Gender**	Male		20 (17.1)	0.062	0.88 (0.30–2.53)	0.807
	Female		16 (29.6)		1 [Reference]	
**Nationality**	Arab		30 (19.5)	0.129	0.58 (0.15–2.31)	0.440
	Non-Arab		6 (35.3)		1 [Reference]	
**Highest degree of education**	No formal education		0 (0.0)	0.834	–	–
	High school diploma		6 (20.0)		–	–
	College or Higher		28 (20.9)		–	–
	Vocational training		2 (33.3)		–	–
**Employment status**	Employed		27 (18.2)	**0.030**	0.60 (0.16–2.28)	0.454
	Not employed		9 (39.1)		1 [Reference]	
**Marital status**	Married		33 (21.2)	>0.999	–	–
	Not married		3 (20.0)		–	–
**Comorbid disease/s**	No		27 (21.3)	>0.999	–	–
	Yes		9 (20.5)		–	–
**Being previously active (attending gym regularly**	Yes		10 (31.3)	0.117	1.72 (0.60–4.97)	0.314
	No		26 (18.7)		1 [Reference]	
**Number of unhealthy dietary change**	None		7 (11.9)	**0.001**	1 [Reference]	
	Yes	1	2 (6.9)		0.38 (0.07–2.12)	0.268
		2	7 (25.0)		1.18 (0.30–4.70)	0.815
		3	5 (26.3)		1.09 (0.22–5.41)	0.917
		4	7(30.4)		2.89 (0.70–11.95)	0.143
		5	8 (61.5)		7.27 (1.58–33.53)	**0.011**
**Mean difference for less controlled**						
**Exercise time difference**^†^**(Hours/day)**			−0.65	**0.001**	0.67 (0.43–1.05)	0.080
**Sitting/reclining time difference**^†^**(Hours/day)**			3.06	**0.020**	0.99 (0.87–1.12)	0.837
**Screen time Difference**^†^**(Hours/day)**			3.75	**<0.001**	1.16 (0.99–1.34)	0.055

Abbreviations: AOR, adjusted odds ratio; CI, confidence interval.

* Variables with P-value <0.250 were included in the multivariable logistic regression model.

## 4 Discussion

This study showed that adults with T2DM experienced some adverse lifestyle changes during the period of home confinement which might result in a rise of diabetes complications and an increase of other non-communicable diseases. The participants reported both favourable and unfavourable dietary changes, reduction in physical activity, increased sedentary behaviours, and weight gain consisting with the results of other studies [12,13]. Persistence of such unhealthy behaviours might lead to obesity and overweight, which in turn can trigger other non-communicable diseases. Moreover, obesity increases the risks of infection from SARS-CoV-2 and is associated with more severe infection and higher chances of hospitalization [14]. As shown in Table 2, adopting unhealthy dietary behaviours was found to be a predictor of weight gain in this study supporting the findings of previous studies [15]. Home-based exercise programs can be safe and effective in breaking the cycle of sedentary behaviours adopted during home confinement measures [16]. About one fifth of participants perceived poorer glyecmic control which might render them vulnerable to diabetic complications. Additionally, the poor glycaemic control can lead to immunosuppression and increase the susceptibility to infections including SARS-CoV 2 [17]. In fact, acute or chronic hyperglycaemia in patients with T2DM which frequently accompanies obesity can impair innate and adaptive immunity by disrupting macrophages function, and enhancing viral replication in monocytes, which can result in secondary T cell dysfunction leading to a more sever clinical course of SARS-CoV 2 in a background of pre-existing chronic inflammation that characterizes both T2DM and obesity [14]. The variability in the perceived glycemic control found in this study as shown in Table 1, reflects the importance of conducting further research with larger sample size that involves actual measurements and follow up of glycemic parameters such as the glycosylated haemoglobin (HbA1C), and fasting blood glucose to draw a conclusion about the real impact of home confinement on the glycemic control.

### 4.1 Strengths and limitations

While this is one the few studies to assess the perceived impact of COVID-19 related home confinement measures on the lifestyle of diabetics in the Middle East, some limitations must be acknowledged. First, relying on the self-reporting by participants to collect data can introduce recall bias. Second, our assessment of physical activity was based on changes in exercise time and other sedentary behaviours such as the sitting/reclining and screen times. However, other types of physical activities such as household work might have been affected so the results should be interpreted with caution. Lastly, the small sample size, and the snowballing sampling might have introduced selection bias and compromised the external validity of the study.

## 5 Conclusion

Further research is needed to explore the persistence of adverse lifestyle changes in the post pandemic era. Diabetics are in need for more intense medical follow ups to restore and optimize their metabolic health after the period of movement restrictions and home confinement.

## Funding

None.

## CRediT authorship contribution statement

**Muna Abed Alah:** Conceptualization, Methodology, Data collection and extraction, Interpretation of data, Date analysis, Project administration, Writing – original draft, Final approval of the version to be submitted. **Sami Abdeen:** Methodology, Data collection and extraction, Interpretation of data,Data analysis, Writing – original draft, Final approval of the version to be submitted. **Vahe Kehyayan:** Interpretation of data, Supervision, Writing – review & editing, Final approval of the version to be submitted. **Iheb Bougmiza:** Interpretation of data, Supervision, Writing – review & editing, Final approval of the version to be submitted.

## Declaration of competing interest

None.
