# Supplementary material for: Impact of COVID-19 related home confinement measures on the lifestyle, body weight, and perceived glycemic control of diabetics
Source: Metabol Open. 2021 Oct 29;12:100144. doi: 10.1016/j.metop.2021.100144 (PMC8553630; doi:10.1016/j.metop.2021.100144)
Supplement: Multimedia component 1 [file mmc1.docx]

**Supplementary File 1:**

**Impact of COVID-19 home confinement measures on the lifestyle and glycemic control of diabetics**

| **Question** | **Choices:** | | | | | |
| --- | --- | --- | --- | --- | --- | --- |
| 1. Age (in years) | If less than 18 years old the survey will end automatically | | | | | |
| 1. Do you suffer from type 2 diabetes mellitus | Yes | | | | No (the survey will end automatically (with a message states the following: **you cannot complete the questionnaire because it is only intended for those who have type 2 diabetes mellitus** | |
| 1. Have you stayed in Qatar for a total duration of at least 2 months in the period between March and August 2020? | Yes | | | | No (the survey will end automatically with a message states the following: **you cannot complete the questionnaire because you have not stayed in Qatar for a total of 2 months in the period between March and August 2020)** | |
| 1. Gender | Male | Female | | | | |
| 1. Nationality | List of nationalities | | | | | |
| 1. Marital status | Married | Not married | | | | |
| 1. Employment status | Employed | Not employed **(skip to question 9)** | | | | |
| 1. Have you worked from home as part of “staying at home” measures since the start of COVID-19 | Yes | No | | | | |
| 1. Highest degree of education you have? | No formal education | High school diploma | | College or Higher | | Vocational training |
| 1. Do you suffer from other chronic disease/s | Yes **(specify)** | | No | | | |

| **Section B: Diet** | | |
| --- | --- | --- |
| 1. **Please choose from the list the changes that describe your eating behavior**   Since the start of COVID-19 pandemic and the imposing of “staying at home” measures: | | |
| I tend to eat more fatty food | Yes | No |
| I tend to eat more sugar/chocolate/sweets | Yes | No |
| I tend to eat more vegetables and/or fruits | Yes | No |
| I tend to eat more fast/junk food | Yes | No |
| I tend to depend more on home cooking for eating | Yes | No |
| I tend to eat more processed/canned food than fresh food | Yes | No |
| I tend to eat larger quantities of food | Yes | No |

| **Section C: Physical activity, sedentary behaviors, and perceived glycemic control** | | | | | | | |
| --- | --- | --- | --- | --- | --- | --- | --- |
| 1. Average time (hours each day) spent in front of TV, computer or mobile (screen time) | Before “staying at home” measures  (___) Hours each day | | | After “staying at home” measures  (___) Hours each day | | | |
| 1. Average time spent sitting or reclining (hours/day) | Before “staying at home” measures  (___) Hours each day | | After “staying at home” measures  (___) Hours each day | | | | |
| 1. Average time spent in exercise (regardless of the type or intensity of exercise) (hours/day) | Before “staying at home” measures  (___) Hours each day | | After “staying at home” measures  (___) Hours each day | | | | |
| 1. Did you use to go to the gym regularly before closure of gyms as part of staying at home measures | Yes | | No | | | | |
| 1. Did you gain weight during “staying at home” measures | Yes | No **(please skip to question 18)** | | | | | |
| 1. On average how many kilograms you gained weight since “staying at home measures” | Less than 3 kg | 3-6 kg | | 7-10 kg | | More than 10 kg | |
| 1. My blood sugar readings since imposing of “staying at home measures” became overall | Less controlled | | | | More controlled | | The same |
| 1. In your opinion, what is/are the reason/s behind less controlled readings (Blood sugar readings)   **(you can choose more than one answer)** | - My diet became less healthy - More sedentary behavior - Limited accessibility to medical services - Limited availability and accessibility to medications - Increase stress and worries (about COVID-19, Financial issues…) - Others, specify | | | | | | |
